# P-919. Significant decline in Vancomycin Use and cost of therapy in Patients with Pneumonia with QI Informatics-based Antibiotic Stewardship Program (ASP) Interventions

**DOI:** 10.1093/ofid/ofaf695.1125

**Published:** 2026-01-11

**Authors:** Ashlesha Kaushik, Brittany Esty, Corey Thieman, Sandeep Gupta

**Affiliations:** UnityPoint Health and University of Iowa Carver College of Medicine, Sioux City, IA; Boston Children's Hospital, Boston, Massachusetts; Unity Point Health at St. Luke's Regional Medical Center, Sioux City, Iowa; Pulmonary and Critical Care Medicine, Unity Point Health, Sioux City, Iowa

## Abstract

**Background:**

According to CDC, vancomycin should be a key-target for ASP; and high negative-predictive-value of nasal MRSA PCR screening (nMP) for pneumonia has been shown.

**Methods:**

Informatics-based ASP interventions were implemented at a tertiary-care-center serving the tristate area in Upper Midwest as current state/ Fishbone analyses showed that vancomycin-use for pneumonia is high and nMP are not being done as not easy to remember/order. SMART aim was to reduce vancomycin-use by 20% for pneumonia by 3/15/25. Automated Default nMP order was incorporated in Pneumonia order-set in EMR (Epic) while ordering vancomycin for Hospital Acquired pneumonia/Ventilator associated pneumonia on 10/7/24 and for Severe Community Acquired pneumonia meeting criteria for vancomycin on 11/4/24.Vancomycin-use during pre-intervention-period(P1: 4/1/24-9/30/24) was compared with intervention-period(P2: 10/15/24-3/15/2025).

**Results:**

Outcome measures (vancomycin duration; cost) showed a significant decline on XmR SPC charts (Figures 1 and 2). Average vancomycin use decreased by 49.5% (from 72.2 DOT/1000 patient days in P1 to 36.3 DOT/1000 patient days in P2, p< 0.01). Average Vancomycin drug inventory cost decreased by 50% (from 1089.5 USD/1000 patient days in P1 to 546.3 USD/1000 patient days in P2; p< 0.05). All Process measures showed a significant, sustained change: proportion of nMP ordering increased to 100% in P2 (p< 0.0001); proportion of negative PCR results leading to discontinuation of vancomycin increased by 84% (p< 0.01) and time to discontinuation of vancomycin declined by >50% (p< 0.05); balancing measures [readmissions and total time from order to PCR results] remained unchanged.

**Conclusion:**

Informatics-based ASP interventions were highly transformative leading to a significant decline in total vancomycin utilization, considerable healthcare-cost savings and significant increase in nMP screening among patients hospitalized with pneumonia; and should be considered for implementation on a wider scale.

**Disclosures:**

All Authors: No reported disclosures

